# Trust Management in Organic Agriculture: Sustainable Consumption Behavior, Environmentally Conscious Purchase Intention, and Healthy Food Choices

**DOI:** 10.3389/fpubh.2019.00340

**Published:** 2019-11-19

**Authors:** George Lazaroiu, Mihai Andronie, Cristian Uţă, Iulian Hurloiu

**Affiliations:** ^1^Faculty of Social and Human Sciences, Spiru Haret University, Bucharest, Romania; ^2^Department of Economic Sciences, Spiru Haret University, Bucharest, Romania

**Keywords:** trust management, organic agriculture, sustainable consumption behavior, environmentally conscious purchase intention, healthy food choices

## Abstract

We draw on outstanding recent research to substantiate factors driving pro-environmental food purchasing behavior. Throwing light on purchasing behavior for environmentally sustainable foods, our study highlights the relevance of consumer trust and motivations in organic product markets together with individuals' perceived value and willingness to buy such items throughout the choice behavior and decision-making process. Our findings prove that most aspects influencing consumers' attitudes for and choices of organic foods are related to their trust and perceptions of the nutritional benefits such products provide. The insights gained from our research extend present knowledge concerning consumer behavior and purchase intention for environmentally sustainable products. The chief gaps and issues identified by the review cover the variety of organic food consumer purchase intentions and behaviors, including the relative environmental performance of organic food production and the link between the motivational values and attitudes concerning the consumption of non-chemical products. Apart from sustainable agriculture and upsides of organic farming, the main disadvantages are as follows: recycling and aligning with natural operations does not necessitate chemical inputs, but organic food is more prohibitive as farmers do not obtain significant crop productivity out of their land, while organic goods may have a price of up to 40% more (production expenses are steeper because farmers demand more labor force), marketing and distribution are not streamlined as organic products are delivered in diminished volumes, food disorders may occur more frequent, and chemical-free agriculture cannot produce sufficient nutrients that the world's population requires to live on.

## Introduction

The purpose of this research is to examine the consistent shift of consumer predispositions and behavior toward food with diminished detrimental health and environmental effects (1), focusing on issues such as consumers' decision-making with reference to organic foods, cognitive and affective attitudes as determinants that shape consumer's impulse to shop for environmentally sustainable products (e.g., the cognitive process underlying additive-free product choices), the link between personal values and opinions in relation to the organic food consumption, and the role consumer trust has in consumer intention to purchase food products having eco-friendly features and certifications, even though certified organic farming is not decisive in the preservation or improvement of the agroecosystem health (2). The insights gained from this review extend present knowledge concerning consumer pro-environmental behavioral attitude and intention as regards organic food purchasing decisions.

The present study provides evidence that safety, nutritional, and health features shape readiness to pay a high price (3) for environmentally friendly certification (4). Communication actions and labeling strategies should be enhanced to boost consumer acceptance and influence relevantly consumer purchase behavior (5). The increase of awareness in the direction of organic food is decisive in stimulating consumers to purchase (6), while they depend on symbolic observations when assessing foods, which may result in distorted judgments and choices (7).

## Consumers' Environmental Attitudes, Sustainable Purchase Intention, and Decision-Making Behavior Regarding Organic Food Products

When shopping for a product, purchasers encounter various information that can only influence their preferences if they consider it (8). Food buying behaviors are shaped by diverse aspects, e.g., health and the environmental consequence of particular food production systems (9). Purchasers' behavioral intentions are determined decidedly by the kind of green product examined and the intrinsic type and scale of participation related to the food category (10). Individual attitude is instrumental in influencing buying intention, up in front of subjective standards and perceived behavioral control (11). Biogas generation on organic farms runs up against numerous structural barriers, hindering their cost-effectiveness. By supplying renewable energy in addition to boosting food outputs and economic soundness, the assimilation of anaerobic digestion in organic farms may generate sustainable or eco-functional build-up of chemical-free agricultural systems (12) to confront the difficult task of productivity increases (13).

Aschemann-Witzel and Zielke (14) insist that accelerating consideration for the environment is accountable for the expanded consumption of non-chemical products. Campaigning for organic farming may enhance sustainability in the food industry, but additional consumer demand is hampered by premium prices that are the chief perceived obstacle to purchase. Khan and Mohsin (15) assert that functional, social, and environmental values favorably influence organic food consumer choice behavior (16), whereas conditional and epistemic values have a negative effect. Functional and emotional values do not shape organic food consumer choice behavior. Chang and Chang (17) show that connection strength and sender's and receiver's environmentally friendly expertise have a beneficial impact on organic consumer's predisposition to informational and regularizing intersubjective influences. The latter both have a constructive relationship on organic buying intention, which thoroughly prevail upon the non-chemical purchase behavior. Wagner Mainardes et al. (18) remark that being predisposed to change constitutes the attitude that may result in the intention to buy additive-free products. The attitude associated with the buying of organic products is an intermediary between the personal values of consumers and their willingness to purchase additive-free goods.

Garcia and Teixeira (19) write that the high price of environmentally friendly products may only be explainable by aspects not including food protection (microbiological soundness and pollutants from green and natural sources are significantly impacted by other elements instead of being entirely unassociated with the production system solely). Reinders and Bartels (20) say that, for both organic private label brands and organic national brands, brand equity thoroughly impacts additive-free brand consumption and non-chemical consumption behavior. For organic private label brands, brand recognition is associated with additive-free brand consumption along with non-chemical consumption behavior. For organic national brands, environmentally friendly consumer identification impacts additive-free brand consumption and non-chemical consumption behavior. The link between brand equity and additive-free brand consumption is somewhat moderated by brand recognition.

Konuk (21) reveal that as health and environmental concerns are gradually relevant in consumers' decision-making process, request for organic food is swiftly increasing. There are beneficial connections between price fairness, contentment, confidence, and purchase intentions with respect to organic food. Juhl et al. (22) explain that individuals embrace organic food products in an unsurprising, nonrandom order, progressively expanding and generalizing their organic buying decision to various product categories over time. By purposefully purchasing non-chemical products, a consumer is likely to galvanize a general personality as an organic food/environmentally responsible buyer, which as a result makes them more predisposed to shop for other kinds of additive-free food. Onel (23) observe that personal and subjective criteria, attitudes in relation to behavior, and intention clarify purchasers' pro-environmental buying behavior, while perceived behavioral control clarifies performance-related intention.

## Consumers' Perceptions, Beliefs, and Trust of the Quality Features of Organic Food

There is a growing body of evidence showing that consumers' values shape their attitude and intention to eat additive-free food (24). Deciding on organic food enables both upgraded environmental effect and enhanced nutritional consequences (25). Environmentally friendly attitudes are positively related to non-genetically modified and organic food ones (26). Sustainable consumption may comprise both sustainable principles and sustainable actions (27). Consumer choice behavior is essential to reinforcing cleaner production and is crucial in energy-saving improvement and green policy-making (28). Enlarging land utilization for vegetables in organic farming shapes the kind of food produced, being monopolized by milk goods and red meat. As crops are reduced under organic procedures, more land is needed to generate the same volume of agricultural yields. A pressure for significant more rural areas determines massive land utilization alterations, generating sweeping environmental effects that arise when converting to organic agriculture (29).

Taufique and Vaithianathan (30) hold that swift economic growth and ensuing overconsumption have intensified green deterioration all over the world, inducing increased consumption-related environmental concerns. Attitudes and perceived purchaser effectiveness both shape favorably ecologically conscious consumer behavior. The subjective criteria, an operation of social demand, cannot shape behavioral intention bringing about ecologically conscious consumer behavior. On Persaud and Schillo (31)'s view, social identity and interactional influence shape purchase intention, while the perceived value of environmentally friendly products somewhat moderates these relationships. Yue et al. (32) stress that to activate the consumer's senses, environmentally friendly food is perceived as cultivated more naturally and rigorously supervised as regards transportation and storage in contrast to conventional products, thus shaping consumers' attitudes and generating their buying interest.

Rana and Paul (33) suggest that demand and supply impediments that adversely impact consumer attitude with regard to additive-free products encompass costs involved to decrease the agricultural chemical utilization, premium price, insufficient availability, and inconvenience in producing organic manure. In the environmentally friendly food market, brand equity significantly shapes the perceived quality and consumer purchase behavior. Environmentally friendly food is an appealing suggestion in a specific market where consumers are health aware and plan to eat safe, wholesome, and organic goods. Pham et. (34) note that encouraging consumer buying behavior of organic foods is essential to environmental sustainability. Food safety issues, health awareness, and media promotion of nutrition information are pivotal in the configuration of attitude concerning non-chemical products, while consumers' environmental sensitivity and meal taste are irrelevant in predicting their attitude. Perceived obstacles, such as premium price, deficient availability, substandard labeling, and additional time demanded, considerably hinder both attitude and purchase intention in relation to organic products. Suciu et al. (35) put it that consumers perceive the organic products more cherished although rather superior prices and scarcer convenience of non-chemical products contrasted with their conventional equivalents may curb the consumption and the quality perception. Golob et al. (36) emphasize that environmentally conscious purchase behavior favorably and environmental disbelief adversely impacts non-chemical food consumption. The former is positively altered by personal and social criteria, perceived convenience, and purchaser sustainability tendency. The social criteria bring into play the most durable indirect effect on environmentally friendly product consumption.

Petrescu et al. (37) state that trust in the certification of the non-chemical products is essential for its purchase. Additive-free food consumers are definitely interested in certified or uncertified organics and oriented by health and taste incentives. The cost of certified organic national goods may be considerably diminished than imported products by cutting down transportation expenses, taxes, and the amount of intermediaries. The rise in the volume of organic producers and of the regions transformed to organic utilization can result in superior output and in a consistent decline of prices, favorably affecting national consumption. Health characteristics, superior taste and a beneficial effect on the environment constitute the essential active components of organic food consumption. Khare and Pandey (38) maintain that environmentally friendly peer impact, perceived organic product quality, and service quality favorably affect perceived trust concerning additive-free food store, while energy-efficient self-identity adversely affects perceived transaction risk. Nuttavuthisit and Thøgersen (39) posit that consumer trust represents a necessary condition for setting up a market for credence products (e.g., premium priced green goods), constituting a definite volition aspect impacting the probability that purchasers will pursue organic intentions. Lack of confidence in the control system and in the naturalness of products marketed as organic adversely influences self-declared purchase behavior. On Ricci et al. (40)'s reading, consumer trust favorably shapes attitudes in relation to the buying of convenience products with green characteristics and detrimentally influences consumer issues regarding agricultural routines as to eco-friendly and health consequences.

Organic crop farmers in underprivileged regions generally do not have adequate access to certification and markets (41). Organic label disallows the utilization of antibiotics, herbicides, fertilizers, and, hormones, but environmental deterioration tends to take place in chemical-free foodstuffs (42). Organic farming is instrumental in preserving a prime health status and cutting down the imminence of developing chronic diseases, because of the abundant amount of bioactive mixtures and inferior volume of detrimental substances, but extensive intervention studies would clarify whether a chemical-free diet is healthier than one encompassing conventionally grown food products (43).

## Does Organic Food Provide Higher Nutritional Benefits than Conventionally Produced Foods?

Health issues are the leading incentive of consumers buying organic products (44). Attitude and health awareness represent important predictors of additive-free food buying intention (45). Healthy and safety food concerns constitute the purchasing process quintessence of environmentally aware consumers (46). The concrete and perceived health and taste ascendancies of environmentally friendly food are primarily derived from the operation of producing food without employing synthetic inputs (47).

Hidalgo-Baz et al. (48) indicate that the organic market involves environmental, health, and food safety issues. Even when expressing favorable attitudes concerning organic foods, consumers often display discordant behaviors and are unsuccessful in purchasing them. Higher knowledge and green tendency of purchasers shape the consonance between their attitudes and buying behavior as regards organic food. Both consumer tendencies displaying the ecological advantages of non-chemical goods and purchasers' knowledge concerning environmentally friendly products are processes that can diminish discrepancies between attitude and shopping behaviors in the additive-free market. Lee et al. (49) find that food firms that promote their goods as additive-free suppose that the organic label convinces consumers that the products are healthy. An organic label may either raise or diminish food consumption being determined by food type and health locus of control. As Husic-Mehmedovic et al. (50) put it, the perceived quality related to the inherent characteristics of organic products moderates a beneficial impact of life balance on consumers' additive-free food purchase intentions. Life balance mediates the repercussions of health awareness on the assessment of inherent and outward food quality features.

Gineikiene et al. (51) show that health-aware consumers are likely to disregard information concerning the health relevance of conventional products and display predispositions for organic food. Reservation as regards health allegations has a more considerable adverse homogenous effect on the perceived healthiness of non-chemical and conventional goods in opposition to health awareness without directly diminishing consumers' inclination to purchase them. Hansen et al. (52) demonstrate that health awareness has a higher beneficial impact on organic product identity with more significant degrees of personal values such as self-transcendence, receptivity to change, self-improvement, and conservation. When receptivity to change is unsatisfactory, health awareness has a beneficial impact on purposeful organic product behavior via non-chemical food identity, while social awareness has a detrimental consequence. Treu et al. (53) observe that, from an ecological perspective, non-chemically cultivated food may not surpass conventional products. Efficiency-increasing initiatives in organic farming are instrumental in diminishing carbon footprints and land utilization. Brantsæter et al. (54) affirm that the growing trendiness of additive-free products has induced the strong interest for organic certification and criteria. If processing of non-chemical food generates ultraprocessed convenience goods, their value of wholeness is damaged. Organic food cultivation and consumption lead to reduced pesticide exposure, are more energy-efficient, and are preferable for animal welfare.

Puska et al. (55) state that when consumers' willingness for status is established, they opt for additive-free food products considerably over their nonorganic equivalents, while making the preference situation observable generates the same outcome. Status reasons and reputational issues lead to an enhanced senso-emotional practice of organic goods. A propensity to advocate organic products is an expensive signifying attribute, resulting in displaying consumers' prosocial predispositions. Underscoring socially criticized consumption reasons (e.g., reputation management) can be a valid manner to boost the somewhat insufficient purchasing of organic products and thus stimulate sustainable consumer behavior. Meyerding and Merz (56) point out that additive-free food products represent credence goods. Organic labels are relevant visual stimuli for consumers to establish how food was produced. Consumers who obtain outstanding utilities from certain characteristics of a product also tackle them visually significantly. On Massey et al. (57)'s account, credence features of organic products are appreciated more than inspection and experience characteristics, having an important role in consumer non-chemical food acquisitions.

Assessment of organic output developed on yield correlations of sets of equivalent crops cultivated non-chemically or using nitrogen fertilizer is unsuccessful in identifying the land that is to be designated to vegetables for biological nitrogen fixation by vegetables to provide nitrogen for the cultivation of non-vegetable crops. The resultant reduced portion of land operational for cereal crops diminishes the entire output of organic agriculture, being unsatisfactorily productive to nourish the present or predictable world population growth (58). Organic crops cultivated in sequences with vegetables or treated with nitrogen manures to a great extent yield not as much as crops cultivated with nitrogen fertilizer, while considerable areas of vegetables are needed to supply sufficient nitrogen for particular yields of non-vegetable crops. By disregarding the consequences of diseases, weeds, and pests, and by neglecting the land needed for vegetables, a substantial exaggeration of the relative output of organic agriculture results (59). Considerable alterations in diet and decreases in food waste cancel out the production consequences of an integral reorganization toward organic farming (60).

## Conclusions

The above literature throws light on purchasing behavior for environmentally sustainable foods. Such empirical evidence supports the belief that organic product labeling can have a pivotal function in decision-making (61) by exerting influence upon health-aware, environmentally concerned individuals and generating store loyalty. Store image has a constructive effect on perceived quality and confidence in organic private label that result in perceived value. Both confidence in organic private label and perceived value have a constructive effect on consumers' purchase intentions. The consequence of perceived quality and confidence in organic private label on purchase intentions is somewhat moderated by perceived value (62). The above studies provide evidence that organic food quality is demonstrated by green, health, and hedonic upsides (63); that inexpensive and abundant products should not affect healthfulness, the ecosystem, and consuming quality (64); that the market is regulated by the perceived upsides of chemical-free over conventionally cultivated food (57); that altruistic value notably influences biosphere values, which subsequently arouse disposition to spend more on an environmentally friendly menu through pro-environmental attitude (65); and that degree of confidence, consumer's additive-free food beliefs, and perceived green accountability favorably affects organic purchase intention (66). The conclusion drawn from these analyses is that organic consumers mainly purchase environmentally friendly products as they think they are healthier and full of nutrients because of the lack of harmful substances (67), as chemical pesticides and synthetic fertilizers are not employed in organic agriculture. Consumers' perceived relevance of wholeness in foods explains their intentions to purchase organic food (68).

Based on an extensive review of recent and relevant literature, our study indicates that organic food production and consumption offer health and sustainability upsides. Current gaps in the research include the consequences of prolonged chemical-free agriculture on soil-derived greenhouse gas emissions, the advancement of circular economic systems, management routines and crops that can adjust to the influence of climate change, bioenergy production associated with organic farming systems, plant breeding and genetics as a manner to substitute chemical inputs, and the use of big data and artificial intelligence in organic agriculture as a groundbreaking way out to cut down agrochemicals and procedures that deteriorate the agroecosystem. Future beneficial research should examine the economic effectiveness of sustainable organic farming hotspots, the nutritional value of organic food products and its subsequent impact on human health, the greenhouse gas emissions and energy efficiencies of organic production systems, the regeneration of biodiversity through environmentally harmonized food production, and decisional aspects motivating farmers to engage in organic agriculture.

## Author Contributions

All authors listed have made a substantial, direct, and intellectual contribution to the work, and approved it for publication.

### Conflict of Interest

The authors declare that the research was conducted in the absence of any commercial or financial relationships that could be construed as a potential conflict of interest.
